# Brain Response Induced with Paired Associative Stimulation Is Related to Repetition Suppression of Motor Evoked Potential

**DOI:** 10.3390/brainsci10100674

**Published:** 2020-09-26

**Authors:** Shohreh Kariminezhad, Jari Karhu, Laura Säisänen, Jusa Reijonen, Mervi Könönen, Petro Julkunen

**Affiliations:** 1 Department of Applied Physics, University of Eastern Finland, 70211 Kuopio, Finland; jusa.reijonen@kuh.fi (J.R.); petro.julkunen@kuh.fi (P.J.); 2 Department of Clinical Neurophysiology, Kuopio University Hospital, 70029 Kuopio, Finland; laura.saisanen@kuh.fi (L.S.); mervi.kononen@kuh.fi (M.K.); 3 Nexstim Plc, 00510 Helsinki, Finland; jari.karhu@nexstim.com; 4 Department of Clinical Radiology, Kuopio University Hospital, 70029 Kuopio, Finland

**Keywords:** repetition suppression, neuroplasticity, transcranial magnetic stimulation, paired associative stimulation

## Abstract

Repetition suppression (RS), i.e., the reduction of neuronal activity upon repetition of an external stimulus, can be demonstrated in the motor system using transcranial magnetic stimulation (TMS). We evaluated the RS in relation to the neuroplastic changes induced by paired associative stimulation (PAS). An RS paradigm, consisting of 20 trains of four identical suprathreshold TMS pulses 1 s apart, was assessed for motor-evoked potentials (MEPs) in 16 healthy subjects, before and following (at 0, 10, and 20 min) a common PAS protocol. For analysis, we divided RS into two components: (1) the ratio of the second MEP amplitude to the first one in RS trains, i.e., the “dynamic” component, and (2) the mean of the second to fourth MEP amplitudes, i.e., the “stable” component. Following PAS, five subjects showed change in the dynamic RS component. However, nearly all the individuals (*n* = 14) exhibited change in the stable component (*p* < 0.05). The stable component was similar between subjects showing increased MEPs and those showing decreased MEPs at this level (*p* = 0.254). The results suggest the tendency of the brain towards a stable state, probably free from the ongoing dynamics, following PAS.

## 1. Introduction

Owing to its dynamicity, the brain responds to an intense, novel stimulus with enhanced, transient neural activity. This rapid response, referred to as a startle, is considered to play a critical function in promoting survival [1]. However, exposure to a higher number of identical sensory stimuli yields attenuation of neural activity in the responding network, a phenomenon known as repetition suppression (RS) [2]. RS has been well-characterized across several brain regions, employing various stimulus categories and modalities [3,4,5,6]. In the motor system, RS has been demonstrated as a decrement in the amplitude of motor-evoked potentials (MEPs) when transcranial magnetic stimulation (TMS) is applied to an optimal motor cortex location [7,8]. Although it has been suggested that the attenuation observed in RS may serve to provide an energy-efficient neuronal information processing [9], the exact mechanisms underlying RS have remained elusive. RS was initially portrayed merely as an expression of bottom-up mechanisms [2,3,10,11]. However, more recent theories have emphasized the role of top–down mechanisms within a predictive coding scheme, relying on iterative comparison between prior expectations and sensory inputs [12]. Interestingly, RS of MEPs have been demonstrated to be closely associated with neuroplasticity [13].

Neuroplasticity is considered one of the key mechanisms that grants living organisms the ability to adapt and respond flexibly in the face of changing environmental demands [14]. Depending on the speed of these changes, neuroplasticity can take different forms and occur at different timescales. Neuroplasticity is considered the keystone of learning, memory, and recovery from (mild) brain injuries [15,16,17]. Aberrant neuroplasticity has been put forth as the pathophysiological basis of several neuropsychiatric disorders, such as schizophrenia, depression, and chronic pain [18,19,20]. Long-term potentiation (LTP) consists of persistent synaptic activity, which is often considered as the cellular basis in the mediation of these functions [21].

Currently, TMS provides the opportunity to study neuroplasticity at the system level, ranging from synaptic plasticity to network-level plasticity [22]. The shifts towards either elevated excitation or diminished inhibition have been proposed as potential underlying mechanisms of neuroplasticity, with the short-term plasticity most likely mediated by the reduction of GABAergic inputs onto excitatory synapses [23].

A well-established and widely used TMS paradigm to induce short-term, topographically specific plasticity in the motor cortex is paired associative stimulation (PAS), in which electrical peripheral nerve stimulation is paired with cortical stimulation [24,25]. If the peripheral input precedes the cortical stimulation, PAS can lead to elevated cortical excitability that manifests itself via an increase in the MEP amplitude (LTP-like plasticity) [26,27]. By contrast, if the order of the arrival of inputs is reversed, depression of cortical excitability is likely to occur (long-term depression (LTD)-like plasticity) [24]. Due to its dependency on timing, PAS has been suggested to induce spike-timing dependent plasticity [28].

In the present paper, to investigate neuroplastic effects induced with PAS, RS is hypothesized to represent the interplay of two states: (1) one reflecting the efficient processing of a novel input, “dynamic RS”, indexed by the initial decrement from the first amplitude to the second one, and (2) one reflecting the overall cortical excitability free from the ongoing dynamics, “stable RS”. Stable RS, described here as the suppressed amplitude level of the second to the fourth MEPs within the RS trials, might potentially display the capacity of the brain to maintain the processed input as an initial “memory trace”. We investigated the dynamic and stable RS prior to and following a common PAS-LTP protocol [24]. We hypothesized that the brain would demonstrate a trend towards a state with low variation in MEP amplitude, which we consider the target level of neuronal network excitability as it is independent from reactive dynamics within the network. As an implication, for long-term neuroplastic effects, the modulation of this stable level could potentially be targeted by neuromodulation, and to create optimal conditions for adaptive neural changes.

## 2. Materials and Methods

### 2.1. Subjects

Sixteen healthy, right-handed volunteers with no history of neuropsychiatric disorders participated in this study (seven male, age range: 22–42 years, mean ± SD: 30 ± six years). All subjects provided a written informed consent prior to the experiment. This study was approved by the research ethics committee of the Kuopio University Hospital (256/2017).

### 2.2. Transcranial Magnetic Stimulation (TMS)

To enable neuronavigation for TMS, structural T1-weighted magnetic resonance images (MRIs) were obtained with a 3T MRI scanner (Philips Achieva 3.0T TX, Philips, Eindhoven, The Netherlands) with the following parameters: repetition time (TR) = 8.2 ms, echo time (TE) = 3.7 ms, flip angle = 8°, voxel size = 1 × 1 × 1 mm^3^. TMS was conducted using NBS System 4.3 (Nexstim Plc, Helsinki, Finland) with an air-cooled figure-of-eight coil and biphasic pulses.

**Figure 1:**
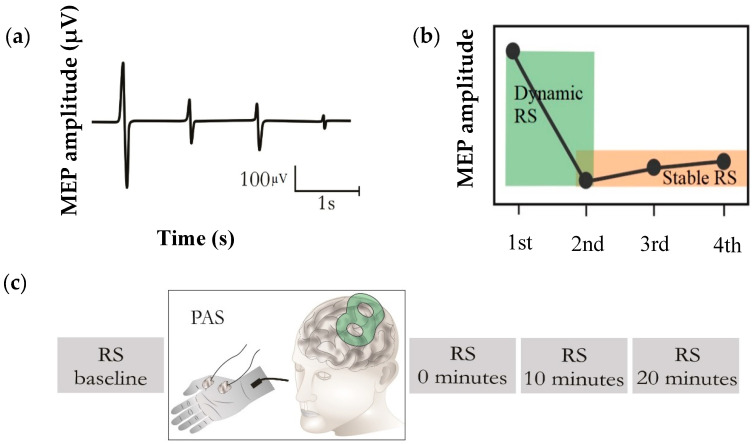
Repetition suppression (RS) paradigm. (**a**) Typical RS. Motor evoked potentials (MEPs) were recorded during four identical TMS pulses. (**b**) The RS paradigm was divided into two components for the analysis: “dynamic RS”, i.e., the ratio of the second MEP to the first one, and “stable RS”, i.e., the mean of the second, third, and fourth MEPs. (**c**) RS applied before (baseline) and after PAS intervention (at 0 min, 10 min, and 20 min). In the PAS intervention, electrical stimulation of the median nerve-innervated APB muscle was delivered prior to TMS at an ISI of 25 ms to generate plasticity.

The stimulation procedure was initiated by locating the optimal motor representation of the right abductor pollicis brevis (APB) muscle, i.e., APB “hotspot”, with the corresponding optimized coil orientation. The hotspot was defined as the cortical site repeatedly eliciting the greatest peak-to-peak MEP responses compared to adjacent stimulation sites. Once the hotspot was determined, the resting motor threshold (rMT) was identified at this cortical site using a system-integrated iterative threshold assessment tool [29]. In the RS paradigm, trials of four TMS stimuli were applied over the APB hotspot, with an inter-stimulus interval (ISI) of 1 s, at an intensity of 120% rMT. The RS paradigm, comprising 20 trials of four single biphasic TMS pulses, was employed with an inter-train interval (ITI) of 17 s [30], before (RS-baseline) and immediately (0 min), 10 min, and 20 min after the PAS intervention (Figure 1).

We recorded MEPs via an integrated electromyography (EMG) system (Nexstim Plc) at a sampling frequency of 3 kHz. A pair of disposable Ag–Cl electrodes was utilized, with the active electrode over the belly of the APB muscle while the reference electrode was placed over the joint distal to the active electrode (Figure 1). The MEP data were processed offline in MATLAB (version R2017b, MathWorks Inc., Natick, MA, USA), and only the MEPs with no preceding muscle activation and peak-to-peak amplitude greater than 50 µV were included as responses.

### 2.3. Paired Associative Stimulation (PAS)

PAS consisting of 180 single stimuli was applied over the right median nerve at an intensity of 300% of the sensory threshold (ST) [31]. A bipolar stimulation electrode was placed over the median nerve, and the ST was measured by adjusting the stimulation current until the subject indicated sensation of the stimulus. The pairing with TMS at the APB hotspot was implemented with a self-built triggering and delayer device. To generate a plasticity effect, median nerve stimulation at a frequency of 0.2 Hz was delivered 25 ms prior to TMS [24], with the TMS pulses delivered at 120% of rMT. The median nerve stimulation was conducted using a constant-current electrical stimulator (Digitimer model DS7A, Digitimer, Welwyn Garden City, Herts, UK), using a rectangular pulse form (0.2 ms, maximum voltage of 300 V).

### 2.4. Statistical Analysis

The MEP amplitudes of each subject were first averaged based on their ordinal position in a trial, i.e., the first, second, third, and fourth. To evaluate the dynamic component of RS, the average of the second stimulus MEP amplitudes was divided by the average of the first stimulus MEP amplitudes. Further, to assess the stable component of RS, the mean of the averaged responses was computed over the second, third, and fourth stimuli per subject.

Considering the inherent heterogeneity of the neurophysiological characteristics, the analysis for identifying significant PAS-effects was initially performed at the individual level using the non-parametric Wilcoxon signed rank test. Individuals with a statistically significant increase in MEP amplitudes at a stable level at 0 min were identified as those showing LTP-like plasticity as an immediate response to PAS (as a higher MEP amplitude is considered as an index of elevated cortical excitability), and clustered as the “LTP-like group”. In addition, individuals with decreased MEP amplitudes at a stable level were considered as those exhibiting LTD-like plasticity as an immediate effect to PAS and clustered as the “LTD-like group”.

To test the change of the dynamic RS and stable RS over a time course of 20 min, the Friedman test was employed. Post hoc comparisons were performed using the Wilcoxon signed rank test.

A comparison of the two clusters prior to and following PAS was made using the Mann–Whitney U test. Statistical analysis was conducted using SPSS (v. 25.0, SPSS Inc., IBM Company, Armonk, NY, USA) and MATLAB (version R2017b, MathWorks Inc., Natick, MA, USA), and *p* < 0.05 indicated statistical significance.

## 3. Results

**Figure 2:**
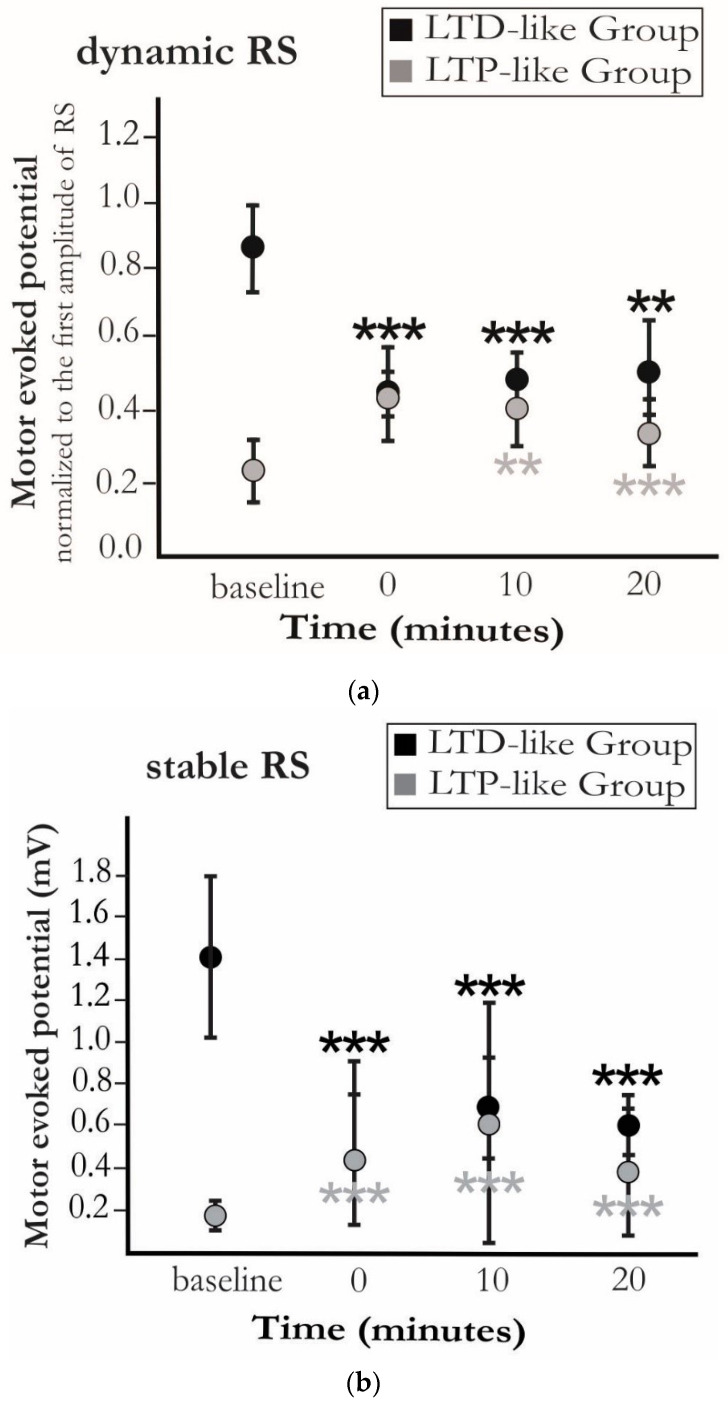
Changes in (**a**) dynamic component (mean ± standard error) and (**b**) stable component of RS (mean ± standard error), across the subjects from two clusters, within the trials before (baseline) and at 0, 10, and 20 min after the induction of short-term plasticity with PAS. Although dynamic RS was mild in the LTD-like group at baseline, a trend towards a stronger suppression and low variability at this level was observed in the LTD-like group following PAS. However, this trend in dynamic RS was towards recovery in the LTP-like group, as this component became milder with time. Similar trends, i.e., sustaining of the suppression and recovery from it, were also observed in stable RS after PAS in the LTD-like group and the LTP-like group, respectively. An asterisk indicates significant differences for pairwise comparisons between time points (*p* < 0.01 for *** and *p* < 0.05 for **).

Eleven subjects showed no significant change of dynamic RS following PAS (*p* > 0.1). Only one subject exhibited significantly milder dynamic RS (a lower drop from the second MEP to the first one) (*p* < 0.05), and four subjects showed significantly stronger dynamic RS (a higher drop from the second to the first MEP) (*p* < 0.05) (Figure 2a).

Fourteen subjects exhibited a significant change at stable RS (Figure 2b). The stable RS levels were significantly higher in six subjects immediately following PAS compared to those before PAS (*p* < 0.05). This heightened post-intervention MEP amplitude was assumed to be linked to LTP-like plasticity. However, eight subjects demonstrated significantly diminished MEP amplitudes at stable RS (i.e., the LTD-like group) (*p* < 0.05), and two subjects showed no significant change (*p* > 0.1). A non-parametric Friedman test revealed no change in the trend over the time of measurement following PAS (0, 10, and 20 min). One subject demonstrated delayed LTP-like plasticity at 20 min after exhibiting no effect at earlier time points.

**Figure A1:**
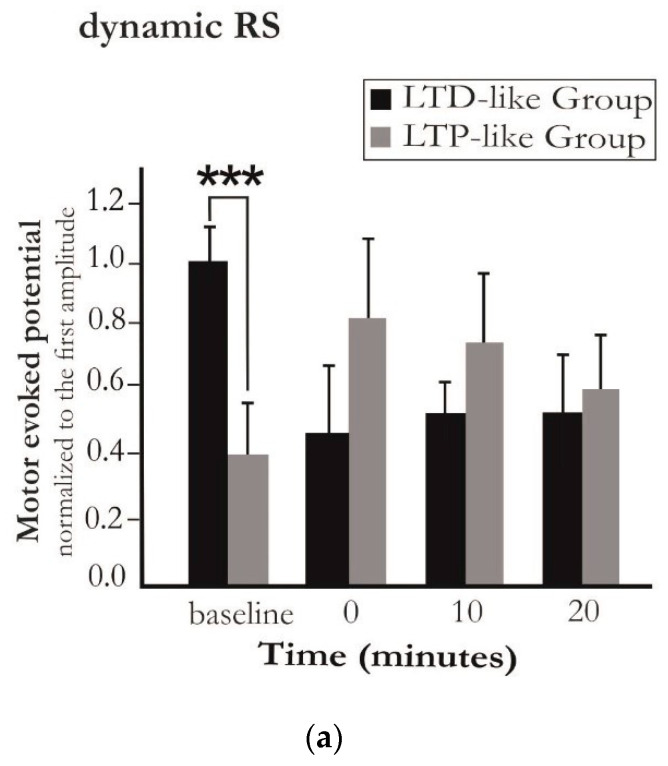

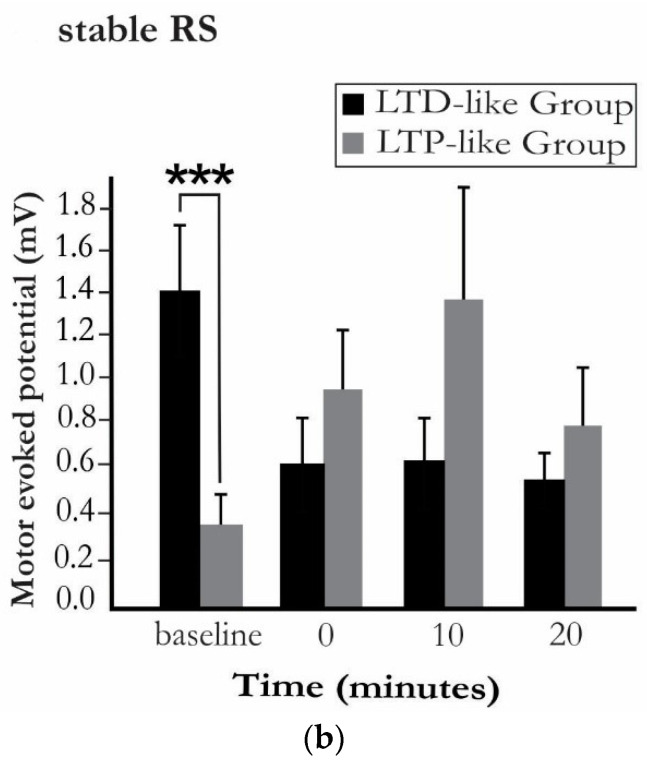
(**a**) Normalized MEP amplitude (mean ± standard error) at dynamic level of RS and (**b**) MEP amplitude (mean ± standard error) at stable level of RS, across the subjects from the LTP/LTD-like groups within the trials before (baseline) and at 0, 10, and 20 min after the induction of short-term plasticity with PAS. Subjects in the LTD-like group exhibited significantly higher pre-intervention MEP amplitudes (baseline) in dynamic RS (*p* = 0.005), and stable RS (*p* = 0.007). No significant difference was observed between the two clusters following PAS in either component. *** indicates significant differences for pairwise comparisons (*p* < 0.01).

Furthermore, a Mann–Whitney U test revealed that the dynamic and stable RS at the baseline was significantly higher in the LTD-like group compared to the LTP-like group (*p* < 0.05). Following PAS, no significant difference in these two components was observed between the two groups (*p* > 0.1) (Figure A1).

The STs were 2.1 ± 0.5 mA, rMTs were 35 ± 8%-maximum stimulator output (MSO) and MEP latencies were 22.8 ± 1.7 ms. No difference in rMT (*p* = 0.845), ST (*p* = 0.244), and MEP latency (*p* = 0.825) was observed between the two groups.

**Figure 3:**
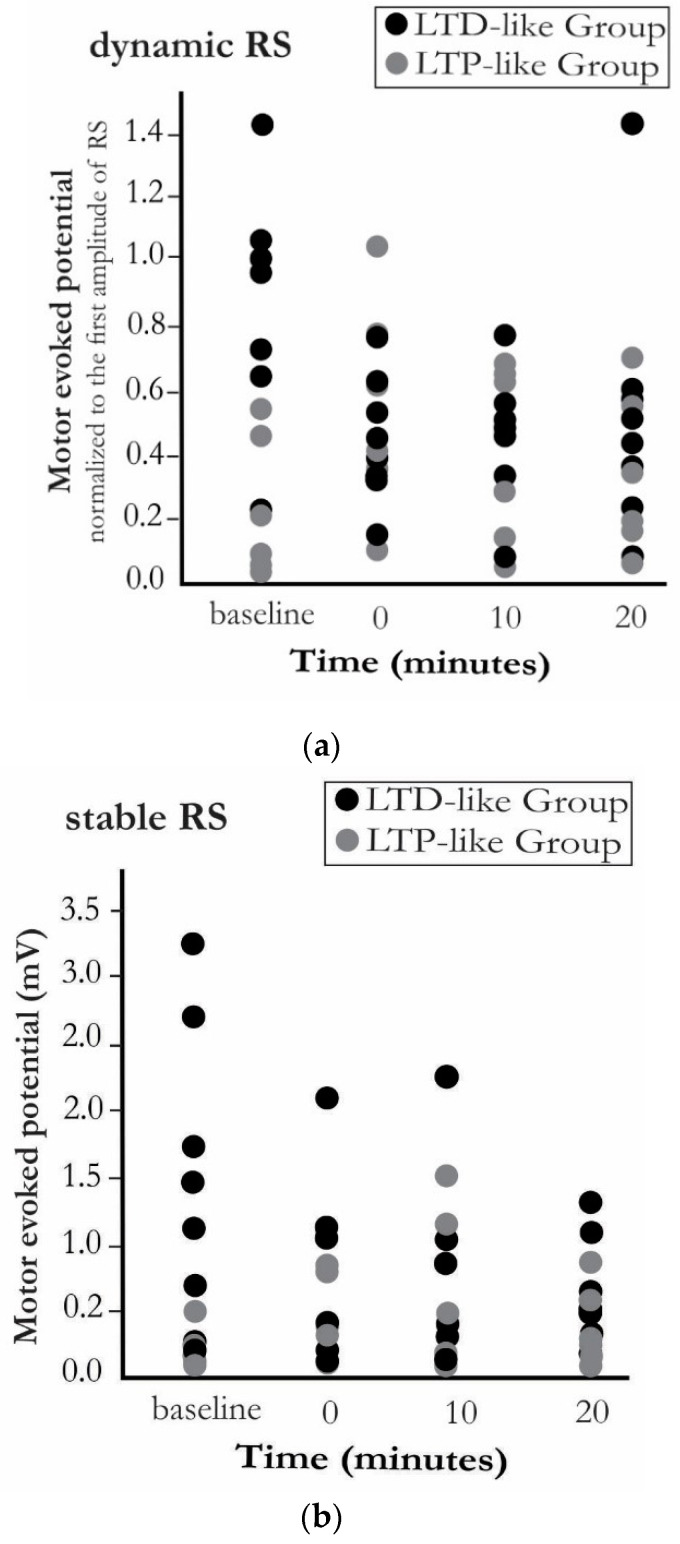
Scatter plot indicating (**a**) the dynamic RS component and (**b**) the stable RS component in all subjects. The low between-group and high within-group homogeneity can be observed in stable RS following applying the PAS intervention, whereas this is not evident for the dynamic RS.

The low between-group and high within-group homogeneities were observed at stable RS prior and following the PAS, respectively (Figure 3).

## 4. Discussion

**Figure A2:**
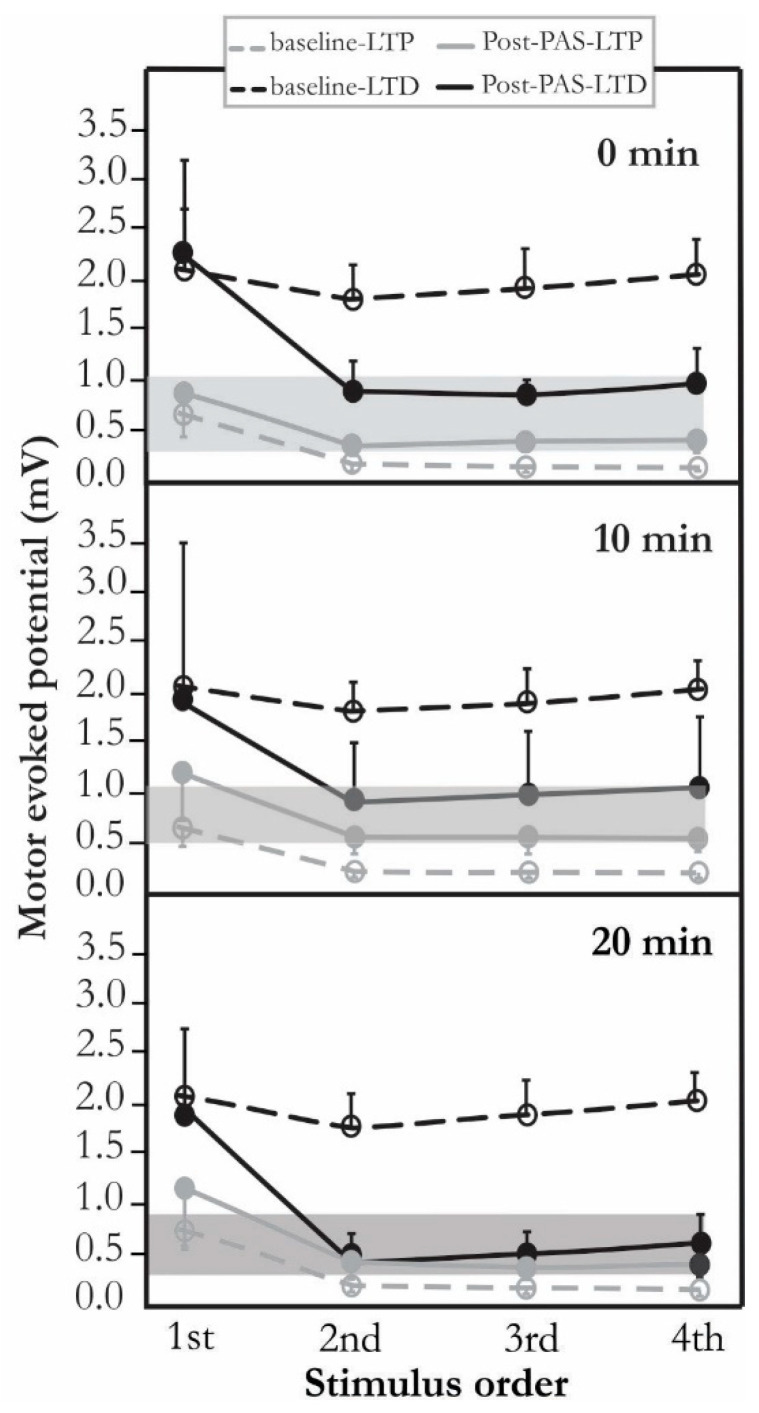
RS at 0, 10, and 20 min after the induction of short-term plasticity with PAS (post-PAS). Effects of LTP/LTD-like plasticity were measured with respect to “baseline” pre-intervention neural activity. Irrespective of the dynamic state of RS, the brain shows a tendency towards a suppressed amplitude level of the second to the fourth MEPs within trials upon repetition of the stimulus. This static status was maintained within a narrow range in two groups after PAS.

Our study investigated two distinct components of RS as a measure of neuroplasticity: (1) immediate changes in motor response upon the first repetition (”dynamic RS”) and (2) the suppressed level of RS (“stable RS”). Surprisingly, induction of plasticity with PAS with a 25 ms ISI resulted in different trends whereof one was rather LTD-like. Irrespective of such a discrepancy, the brain demonstrated an overall tendency towards a common level in stable RS following PAS intervention (Figure A2).

Minimizing the surprise encountered in the face of a novel stimulus is the principle behind the free energy principle [12]. According to this principle, to maintain its integrity, any adaptive biological system, like the brain, seeks to minimize its free energy [32]. It has been proposed that minimizing the free energy rests on either changing the top–down predictions, which are the conceptual internal models, or the bottom–up predicted sensory inputs [32]. In this regard, the dynamic RS depicts an update of the predictions in response to a twice repeated stimulus, ensuring an efficient sensory processing in a known environment. In other terms, the attenuation of the stimulus-evoked motor response upon the first repetition of the stimulus reduces the prediction errors originating from the incoming sensory information. In this respect, the suppressed level of the MEPs during the stable, suppressed part of RS may reflect a level of cortical excitability that is relatively free from ongoing dynamics in cortical excitability, which exhibits as a characteristically high intra-individual variance in the MEPs and may affect the sensitivity of MEPs to reveal longitudinal changes in excitability due to long-term neuromodulation and -plasticity.

RS has been demonstrated to last over short timescales in the visual and auditory systems, indicating a memory trace of the recently viewed or heard stimulus [33]. This short-term storage of information is reflected in our findings in the stable RS. A potential explanation for the observed stable RS might go back to the existence of a short-term internal representation of the perceived involuntary movement (“automatic memory”). Evidence consistent with this postulate is the lack of RS while an ITI of less than 3 s was employed in a TMS study, with the RS being more pronounced with longer ITIs [30]. Apart from the initial motor response, the subsequent responses elicited by TMS are modulated by sensory feedback, i.e., their magnitudes are controlled by the sensory inputs onto the motor neurons. The brain embodies a dynamic interconnected hierarchal processing organization that enables the reciprocal influence of current and past information. A plethora of positive and negative-feedback connections at both the cellular and network levels is central to sustaining the encoded sensory information on a timescale of seconds [34,35]. Hence, to maintain the automatic memory over a short timescale in RS, a negative feedback probably needs to be provided via recruiting inhibitory pathways to sustain the underlying neural activity. These pathways include the intracortical sensory areas and subcortical areas, among which the basal ganglia and thalamus play a key role. It has been demonstrated via RS that this stable state cannot be achieved in patients with progressive myoclonus type 1, who have impaired neuroplasticity in the thalamo-cortical connections [13,36].

Both dynamic and stable RS might reflect alterations in synaptic efficacy. The persistent changes in synaptic efficacy serve as a window into the formation of synaptic plastic changes, a candidate mechanism through which PAS works [26]. If the neuronal network is provided with only a positive feedback loop, that is, the spiking activity of the presynaptic neuron is correlated with the spiking activity of the postsynaptic neuron, its stability gets disrupted. In fact, this unidirectional process reduces the threshold for the presynaptic neuron to stimulate the postsynaptic neuron, thus precluding the stability and reversibility of the system. To counteract this instability and to tune the neuronal activity within a functional dynamic boundary, the brain employs an array of homeostatic mechanisms [37]. Homeostatic plasticity provides the necessary negative feedback loop to prevent the neural circuits from hyper- or hypo-activity.

A well-established proposed mechanism for homeostatic plasticity is the Bienenstock, Cooper, and Munro (BCM) model [38]. This model assumes a bidirectional synaptic plasticity, where the threshold for LTP/LTD induction varies as a function of the dynamic state of the brain. Considering this model, the more excitable the corticospinal pathway is, the more capacity for inhibition may be required. This can in part explain the reversal of the LTP-like plasticity effect to LTD-like plasticity in individuals showing higher pre-intervention MEP amplitudes (baseline). The degree of the modifications of neuronal plasticity depends on updating the synaptic efficacy. Thus, assessing the RS in the mentioned terms, i.e., are dynamic RS and stable RS, can provide us with information on how the alteration in synaptic efficacy following PAS can be reflected in RS.

A few limitations need to be acknowledged in this study. First, we applied the PAS paradigm using a fixed ISI of 25 ms. Inter-individual variability in responses has been reported for PAS due to non-optimized timing of the peripheral stimulus [39]. The potential decrease in this variability might have been achieved by employing an individualized ISI [40]. However, we did not measure individual sensory evoked potential to optimize PAS for LTP-like effects. This was by design to enable more inter-individual latency variance in the induced PAS effect, and to make the sessions shorter for the subjects. Second, in spite of having a sample size within the range of other studies in this field, the number of subjects was still small to account for generalization in large populations or in patients. We consider this a successful proof-of-concept study, but for application in patient groups, a larger-scale trial is required considering more inter-individual heterogeneities. Thirdly, we identified the PAS effect from the suppressed responses of RS (stable RS) to avoid the dynamicity of causing variance in the identification of the plastic effects, as we observed in the case of the first responses in the RS trials. This is not common practice with PAS. However, since no previous studies have been conducted with PAS in relation to RS, we had no point of reference. 

## Appendix A

Figure A1, Comparison of LTD-like group and LTP-like group prior (baseline) and following PAS (at 0, 10, and 20 min).

Figure A2, RS at 0, 10, and 20 min after PAS in LTD-like and LTP-like groups.

## References

[1] Moruzzi G., Magoun H.W. (1949). Brain stem reticular formation and activation of the EEG. Electroencephalogr. Clin. Neurophysiol..

[2] Grill-Spector K., Henson R., Martin A. (2006). Repetition and the brain: Neural models of stimulus-specific effects. Trends Cogn. Sci..

[3] Desimone R. (1996). Neural mechanisms for visual memory and their role in attention. Proc. Natl. Acad. Sci. USA.

[4] Miller E.K., Li L., Desimone R. (1993). Activity of neurons in anterior inferior temporal cortex during a short-term memory task. J. Neurosci..

[5] Krekelberg B., Boynton G.M., van Wezel R.J.A. (2006). Adaptation: From single cells to BOLD signals. Trends Neurosci..

[6] Näätänen R., Picton T. (1987). The N1 Wave of the Human Electric and Magnetic Response to Sound: A Review and an Analysis of the Component Structure. Psychophysiology.

[7] Löfberg O., Julkunen P., Tiihonen P., Pääkkönen A., Karhu J. (2013). Repetition suppression in the cortical motor and auditory systems resemble each other—A combined TMS and evoked potential study. Neuroscience.

[8] Löfberg O., Julkunen P., Pääkkönen A., Karhu J. (2014). The auditory-evoked arousal modulates motor cortex excitability. Neuroscience.

[9] Friston K., Kilner J., Harrison L. (2006). A free energy principle for the brain. J. Physiol. Paris.

[10] Li L., Miller E.K., Desimone R. (1993). The representation of stimulus familiarity in anterior inferior temporal cortex. J. Neurophysiol..

[11] Sobotka S., Ringo J.L. (1996). Mnemonic responses of single units recorded from monkey inferotemporal cortex, accessed via transcommissural versus direct pathways: A dissociation between unit activity and behavior. J. Neurosci..

[12] Friston K. (2005). A theory of cortical responses. Philos. Trans. R. Soc. Lond. B. Biol. Sci..

[13] Julkunen P., Löfberg O., Kallioniemi E., Hyppönen J., Kälviäinen R., Mervaala E. (2018). Abnormal motor cortical adaptation to external stimulus in Unverricht-Lundborg disease (progressive myoclonus type 1, EPM1). J. Neurophysiol..

[14] Pascual-Leone A., Amedi A., Fregni F., Merabet L.B. (2005). The plastic human brain cortex. Annu. Rev. Neurosci..

[15] Rioult-Pedotti M.-S., Friedman D., Hess G., Donoghue J.P. (1998). Strengthening of horizontal cortical connections following skill learning. Nat. Neurosci..

[16] Stefan K., Wycislo M., Gentner R., Schramm A., Naumann M., Reiners K., Classen J. (2006). Temporary occlusion of associative motor cortical plasticity by prior dynamic motor training. Cereb. Cortex.

[17] Rossini P.M., Dal Forno G. (2004). Neuronal post-stroke plasticity in the adult. Restor. Neurol. Neurosci..

[18] Daskalakis Z.J., Christensen B.K., Fitzgerald P.B., Chen R. (2008). Dysfunctional neural plasticity in patients with Schizophrenia. Arch. Gen. Psychiatry.

[19] Player M.J., Taylor J.L., Weickert C.S., Alonzo A., Sachdev P., Martin D., Mitchell P.B., Loo C.K. (2013). Neuroplasticity in depressed individuals compared with healthy controls. Neuropsychopharmacology.

[20] Flor H. (2003). Cortical reorganisation and chronic pain: Implications for rehabilitation. J. Rehabil. Med..

[21] Voronin L.L. (1983). Long-term potentiation in the hippocampus. Neuroscience.

[22] Pascual-Leone A., Tarazona F., Keenan J., Tormos J.M., Hamilton R., Catala M.D. (1999). Transcranial magnetic stimulation and neuroplasticity. Neuropsychologia.

[23] Chen R., Cohen L.G., Hallett M. (2002). Nervous system reorganization following injury. Neuroscience.

[24] Stefan K., Kunesch E., Cohen L.G., Benecke R., Classen J. (2000). Induction of plasticity in the human motor cortex by paired associative stimulation. Brain.

[25] Tolmacheva A., Savolainen S., Kirveskari E., Brandstack N., Mäkelä J.P., Shulga A. (2019). Paired associative stimulation improves hand function after non-traumatic spinal cord injury: A case series. Clin. Neurophysiol. Pract..

[26] Wolters A., Sandbrink F., Schlottmann A., Kunesch E., Stefan K., Cohen L.G., Benecke R., Classen J. (2003). A temporally asymmetric Hebbian rule governing plasticity in the human motor cortex. J. Neurophysiol..

[27] Stefan K., Kunesch E., Benecke R., Cohen L.G., Classen J. (2002). Mechanisms of enhancement of human motor cortex excitability induced by interventional paired associative stimulation. J. Physiol..

[28] Müller-Dahlhaus F., Ziemann U., Classen J. (2010). Plasticity resembling spike-timing dependent synaptic plasticity: The evidence in human cortex. Front. Synaptic Neurosci..

[29] Awiszus F., Borckardt J. (2012). TMS Motor Threshold Assessment Tool 2.0. http://clinicalresearcher.org/software.htm.

[30] Pitkänen M., Kallioniemi E., Julkunen P. (2017). Effect of inter-train interval on the induction of repetition suppression of motor-evoked potentials using transcranial magnetic stimulation. PLoS ONE.

[31] Hamada M., Strigaro G., Murase N., Sadnicka A., Galea J.M., Edwards M.J., Rothwell J.C. (2012). Cerebellar modulation of human associative plasticity. J. Physiol..

[32] Friston K. (2010). The free-energy principle: A unified brain theory?. Nat. Rev. Neurosci..

[33] Ranganath C., Rainer G. (2003). Neural mechanisms for detecting and remembering novel events. Nat. Rev. Neurosci..

[34] Lim S., Goldman M.S. (2013). Balanced cortical microcircuitry for maintaining information in working memory. Nat. Neurosci..

[35] Frank M.J., Loughry B., O’reilly R.C. (2001). Interactions between frontal cortex and basal ganglia in working memory: A computational model. Cogn. Affect. Behav. Neurosci..

[36] Koskenkorva P., Khyuppenen J., Niskanen E., Könönen M., Bendel P., Mervaala E., Lehesjoki A.E., Kälviäinen R., Vanninen R. (2009). Motor cortex and thalamic atrophy in Unverricht-Lundborg disease: Voxel-based morphometric study. Neurology.

[37] Turrigiano G.G., Leslie K.R., Desai N.S., Rutherford L.C., Nelson S.B. (1998). Activity-dependent scaling of quantal amplitude in neocortical neurons. Nature.

[38] Bienenstock E.L., Cooper L.N., Munro P.W. (1982). Theory for the development of neuron selectivity: Orientation specificity and binocular interaction in visual cortex. J. Neurosci..

[39] López-Alonso V., Cheeran B., Río-Rodríguez D., Fernández-del-Olmo M. (2014). Inter-individual variability in response to non-invasive brain stimulation paradigms. Brain Stimul..

[40] Campana M., Papazova I., Pross B., Hasan A., Strube W. (2019). Motor-cortex excitability and response variability following paired-associative stimulation: A proof-of-concept study comparing individualized and fixed inter-stimulus intervals. Exp. Brain Res..

